# Determinants of hand grip strength among community dwelling older adults in southwestern Nigeria: Data from the VALIANT Study

**DOI:** 10.1002/alz.093554

**Published:** 2025-01-09

**Authors:** Rufus O. Akinyemi, Oludotun V Olalusi, Tolulope O Akinyemi, Joseph O Yaria, Gabriel O Ogunde, Joshua O. Akinyemi, Brian Lawlor, Adesola Ogunniyi

**Affiliations:** ^1^ Neuroscience and Aging Research Unit, Institute of Advanced Medical Research and Training, College of Medicine, University of Ibadan, Ibadan Nigeria; ^2^ Africa Dementia Consortium, Ibadan, Ibadan Nigeria; ^3^ University College Hospital, Ibadan, Oyo Nigeria; ^4^ Lead City University, Ibadan, Oyo Nigeria; ^5^ College of Medicine, University of Ibadan, Ibadan, Oyo Nigeria; ^6^ Department of Epidemioloy and Medical Statistics, College of Medicine, University of Ibadan, Ibadan Nigeria; ^7^ Global Brain Health Institute, Trinity College Dublin, Dublin Ireland; ^8^ Trinity College Institute of Neuroscience, School of Psychology, Trinity College Dublin, Dublin Ireland; ^9^ College of Medicine, University of Ibadan, Ibadan, Oyo State Nigeria

## Abstract

**Background:**

Hand‐grip strength (HGS) is known to be a surrogate marker of not only fitness and frailty, but of cognitive and cardiometabolic health. It is cheap, readily deployed and can be a valuable tool in resource‐limited settings. Little however is known about the determinants and correlates of HGS in sub‐Saharan Africa, where stroke and vascular cognitive disorders are projected to exponentially increase. We examined the determinants of HGS among older adults in a rural community in Ibadan, South West Nigeria.

**Method:**

Vascular heAlth, fraiLty and cognition in Ageing Nigerians (VALIANT) Study is an ongoing longitudinal community‐based cohort study aimed at exploring the association between cardiovascular health, cognition and frailty in Nigeria. One thousand (1000) participants have so far been recruited (via a multistage, stratified cluster random sampling method) and have been taken through a battery of cardiovascular, cognitive and frailty assessment tools. Data on HGS, obtained using a digital hand dynamometer, was available for 480 men and women aged ≥50years. A multivariable adjusted linear regression analysis was used to assess the determinants of HGS. All associations were reported as coefficients with 95% confidence intervals (CI)

**Result:**

The mean age was 64.5 (11.8) with 35% males. The mean HGS was higher among males (22.86 10.1) than in females (16.26 6.1) (p<0.001) and decreased with increasing age and in the left hand. Using the Rockwood frailty scale, 66 (13.8%) of the study participants were vulnerable to frail while 80% had cognitive impairment (MoCA <26). In the multivariable linear regression analysis, the independent determinants of hand grip strength with corresponding beta coefficients (95%CI) were attainment of tertiary/postgraduate education 5.19 (1.70; 8.68), being a widow/widower ‐2.75 (‐5.47; ‐0.03), lower MoCA score<19 ‐2.50 (‐4.42; ‐0.59) and higher IDEA‐IADL score 0.23 (0.02, 0.44)

**Conclusion:**

Amongst older adults in rural Nigeria, attainment of tertiary/postgraduate education was independently associated with higher HGS; while being a widow/widower and presence of low cognitive reserve were independently associated with lower HGS. This study has identified unique determinants of HGS among West Africans.

